# Comparision of Barry and Barry-Taguchi Ureterovesical Reimplantation Techniques in Kidney Transplantations: A Randomized Clinical Trial

**Published:** 2010-05-01

**Authors:** M. R. Mohammadi Fallah, A. Taghizadeh Afshari, M. Asadi, A. H. Sharafi

**Affiliations:** Urology and Nephrology Research Center, Urmia University of Medical Sciences, Urmia, Iran

**Keywords:** Kidney transplantation, Ureteroneocystostomy, Ureteral stenosis, Urinary leakage, Hematuria

## Abstract

Background: Renal transplantation is the treatment of choice for chronic renal failure. Using a suitable ureterovesical anastomosis technique can prevent most of risks for kidney graft. Extravesical ureteroneocystostomy is becoming popular in renal transplantation because of the low complication rate and technical ease. The decreased complication rate is due to limited bladder dissection and the need for a shorter ureteral segment from the donor.

Objective: In this study we assessed the effectiveness and complications of a new technique, Barry-Taguchi technique and compared it with Barry technique.

Methods: We recorded all urological complications developed in the recipient’s kidney between September 2004 and March 2007 (mean follow-up 12 months) after performing extravesical Barry-Taguchi (new technique) and Barry ureteroneocystostomy. The urological complications studied included complicated hematuria, urinary fistula, and ureteral stenosis.

Results: A total 100 patients who underwent Barry-Taguchi technique and 98 patients who underwent Barry technique were studied. The incidence of urological complications in Barry-Taguchi and Barry re-implantation technique was 4% (n=4) and 5% (n=5%), respectively. These complications included 1 urinary leakage and 3 ureteral obstructions for Barry-Taguchi technique, and 4 obstructions and 1 leakage from Barry group. In both trial groups, no complicated hematuria has occurred. In addition, the recorded time taken for ureteral anastomosis ranged from 4 to 16 (mean 8.3) min for Barry-Taguchi technique and 5 to 20 (mean 9.9) min in Barry technique.

Conclusion: The Barry-Taguchi extravesical ureteroneocystostomy technique is a rapid and rather simple technique. Without increasing the incidence of urological complication rate, it is a reliable method for performing ureteroneocystostomy.

## INTRODUCTION

The treatment of choice for patients with end-stage renal disease is renal transplantation. Despite improvements in peritoneal dialysis and hemodialysis, these patients survive much longer after receiving a kidney transplant. Survival rates have improved because of refined surgical techniques, more effective immune-suppression with medications such as cyclosporine and OKT3, improved availability of human leukocyte antigen typing for donor-recipient matching, and establishment of a nationwide coordinating network. These advances have enabled a better quality of life for these patients.[1]

Currently, one in every four patients with end-stage renal disease in the US is a kidney transplant recipient.[1] A considerable part of this success is due to the better short-term outcomes that have resulted from improvements in the management of early post-surgical complications and the successful prevention of acute rejection episodes.[2]

The urological complications that occur after renal transplantation have been addressed in many reports. With the current improvements in graft survival plus availability of better immunosuppressant drugs, it would be unacceptable to lose a patient or graft as a result of technical issues. This is important when dealing with living donors.[1]

Extravesical ureteroneocystostomy is becoming popular in renal transplantation because of its low complication rate and technical ease.[2-5] The decreased amount of these complications was due to limited bladder dissection and the need for a shorter ureteral segment from the donor.[6-10].

In our center, Lich Gregory technique had been used for several years with results similar to those reported by others.[11] Since 1993, we have switched to the extravesical technique described by Barry, because it has been proved to be simpler and successful. We have used Barry-Taguchi (new technique) for ureteral reimplantation since 2003. 

We conducted this study to compare Barry and Barry-Taguchi techniques based on the hypothesis that the complication rate in the latter technique is similar to that of Barry technique in urinary tract reconstruction, but it is much simpler and more rapid in renal transplantation.

## MATERIAL AND METHODS

In a prospective randomized study conducted between September 2004 and March 2007, the Barry-Taguchi and Barry extravesical ureteroneocystostomy techniques were preformed on 198 kidney transplant recipients. We randomized patients by a computer program into two treatment arms. The new technique and its potential complications and outcomes were completely explained for patients and if he/she accepted it, formal consent was obtained. The data were obtained by a urology resident after following the patients. Data were analyzed by a statistician who was blind to the group allocations, donor and recipient age and gender, type and time of ureteral implantation, urological complications, and need to reoperation or nonsurgical interventions. We defined urological complications as 1) urinary leak, 2) ureteral stenosis requiring temporary radiological or permanent surgical interventions, 3) ureteral fistulae, and 4) complicated hematuria.

Barry re-implantation technique was previously described; [12] Barry-Taguchi technique will be described. Ureteral stents were used in all cases. Two transplant surgeons performed all the ureteroneocystostomies using the pre-assigned techniques regardless of bladder or ureteral quality. Means of two normally-distributed continuous variables were compared by two-tailed unpaired Student’s t test. Proportions were compared by ϰ^2^ test. All data is presented as mean or median. A P value <0.05 was considered statistically significant.

All patients had a follow-up at the center for at least one year after the surgery. After the renal transplantation, the recipients were monitored clinically and biochemically. When a urological complication was suspected, multiple modalities were employed for diagnosis, including ultrasonography, intravenous urography, antegrade pyelography, retrograde cystography and CT scan. All urological complications were treated as soon as possible by endourologic intervention or open surgery. This method of surgery was used in one previous study with no major complications and with reasonable success. This study was approved by Urumia University Institutional Ethics Committee.

Technique

After anastomosis of the renal vessels to the recipient’s circulatory system, attention was directed to the donor’s ureter in order to confirm that there is adequate blood supply. The kidney was positioned, and the ureter was cut to necessary length. The end of the ureter was spatulated approximately 0**.**5 cm being certain the spatulation does not damage the periureteral vessels. After this, we inserted a double pig-tail stent into the ureter.

The Bladder was gravity filled with antibiotic solution and catheter tubing was clamped next to the drainage bag. After removing the fibro-fatty tissue from the bladder, it was rotated interiorly. 

Two parallel, horizontal incisions were cut through the bladder 3 to 4 cm apart. The distal incision was extended down to the bulging mucosa. Extravesical sub-mucosal tunnel was created with right angle clamp. Stented and spatulated transplant ureter was grasped with right angle forceps and pulled through sub-mucosal tunnel. Nurse released clamp and drained antibiotic solution from bladder. Horizontal incision was made in bladder mucosa (this part is similar in both groups).

**Figure 1:**
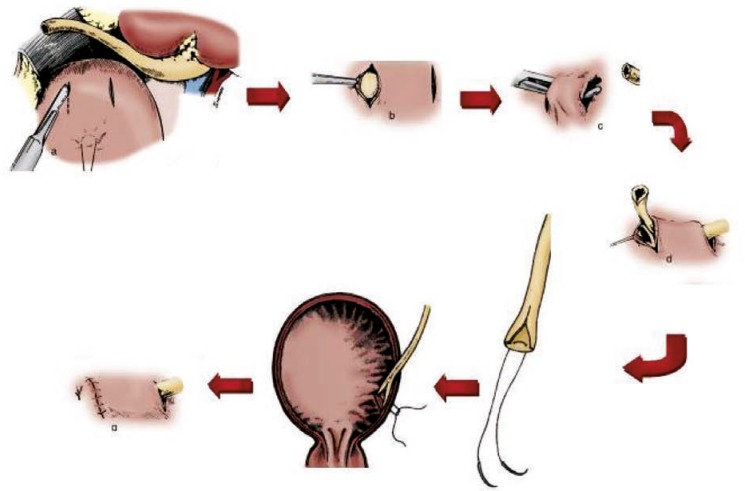
Schematic drawing of Barry-Taguchi ureteroneocystostomy technique

The ureter in the Barry technique was sutured to the bladder using three stitches from the apex and laterals of spatulated segment ureter to the bladder mucosa and one U-shaped suture to the bladder’s wall, while in the Barry-Taguchi technique only one U-form suture was used for re-implantation of ureter (such as Taguchi). We used only one needle suture. The needle of Vicryl 4-0 was passed through the bladder wall and extended through the new orifice. This needle passed continuously through outside to inside and inside to outside of the donor’s ureter. At the end, the needle that was passing through the new orifice also extended through the wall of the bladder. The two ends of the suture should be approximately 1 cm apart from each other, and 2 cm from the new orifice as they exit from the bladder. The ends of the sutures were then gently snagged to the bladder wall and tied (Fig. 1). The distal cystostomy was closed with interrupted 3-0 Vicryl suture. Ureteral stent was removed after two weeks.

## RESULTS

We included 198 cases (100 cases in the group of Barry-Taguchi and 98 cases in the group of Barry technique) in the study.

The mean age of kidney recipients in the Barry-Taguchi group was 36.3 (range: 6–64) years and in the Barry group was 37.8 (range: 13–67) years (P=0.468). In Barry-Taguchi and Barry technique groups, the male:female ratio recipients was 52.8:47 and 57.1:42.9 (P= 0.558). There was no significant difference between the distribution of age and gender in these two groups.

A total of nine (4.5%) transplants which required surgical exploration, were associated with urological complication within one year after the transplantation (Table 1). The incidence of urological complications in Barry-Taguchi and Barry re-implantation technique was 4% (n=4) and 5% (n=5), respectively (P=0.710). Clinical signs and symptoms of urological complications that were observed after the transplantation included 1) increasing serum creatinine or clinical signs of urinoma and obstruction; 2) urine leakage through wound; 3) hemturia; and 4) urinary tract infection (UTI).

**Table 1:** Comparing complications in the two studied groups of patients

**Complication**	**Barry-Taguchi (n=100)**	**Barry (n=98)**	**P value **
Complicated hematuria	0 (0%)	0 (0%)	0.999
Obstruction	3 (3%)	4 (4%)	0.781
Urinary leakage	1 (1%)	1 (1%)	0.989
Overall	4 (4%)	5 (5%)	0.710

In Barry-Taguchi re-implantation technique three (3%) and in Barry re-implantation technique four (4%) patients were suspected to have obstruction; in comparison, no difference was found between the two groups (P=0781). Ultra-sonography found moderate to severe hydronephrosis. Percutaneous nephrostomy was performed in these patients. In antegrade pyelography or surgical exploration, ureteral stenosis at the vesicoureteral anastomosis site was the major complication. Endoscopic and conservative treatments for these patients were not successful. In two cases, ureteroureterostomy was performed using donor and native ureter; in another two cases, pyeloureterostomy was performed using donor pelvis and native ureter; in one patient, vesicopyelostomy was performed; and in the last case, second re-implantation was preformed. One patient with obstruction in Barry group died of sepsis and coagulopathy and probably disseminated intravascular coagulopathy (DIC).

One patient from each group developed urine leakage because of a defect in the bladder and ureter anastomosis site. From our comparison, we found no significant difference in the leakage rate between the two groups (P=0.989). These patients did not respond to conservative and endoscopic treatments. They required surgical revision of the anastomosis and closure of the bladder.

Complicated hematuria that requires removal of the blood clot with endoscopy or reoperation did not occur in our trial. Bladder washing with urethral catheter for clot was required in four (4%) patients in Barry-Taguchi group and in three (3%) in Barry group (P=0.781).

The mean follow-up after the primary transplant was 12 (range: 6–24) months. Multivariate analysis showed that neither surgical treatment nor urological treatment of urological complications increased the risk of overall graft failure or death.

Time of ureteral anastomoses ranged from 4 to 16 (mean 8.3) minutes in Barry-Taguchi and 5 to 20 (mean 9.9) minutes in Barry. As a result, we showed significantly less time consumption by Barry-Taguchi technique in our hands.

## DISCUSSION

Since the advance of kidney transplantation, urological complications have been a major concern. In early reports of renal transplantation, the prevalence of urologic complications varied from 10% to 25%, with a mortality rate ranging from 20% to 30%.[13-14] In these patients ureteroureterostomy or pyeloureterostomy was used to restore urinary tract continuity. Approximately two-thirds of the early urologic complications (urine leak, fistula formation, or obstruction) were apparent in the first month after transplantation and were treated by the transplantation team. Currently, urologic complication rates are 4%–8% with very low patient mortality rate.[13-14]

The urological complications are due to technical problems or ischemia in the early phase. However, ischemia and infection are more common in the later phases.[12] Reports that described low rates of complications revealed several underlying causes including routine use of a ureteral stent, shorter ureteral length, and increased surgical experience and skills.[15]

Extravesical ureteroneocystosomy has slowly gained popularity since its description by Lich in 1961 as a method for ureteral reimplantation in kidney transplant recipients. Complication rates of 3.7% to 7.5% for other methods of extravesical ureteroneocystosomy have been reported by several centers.[4-6,16] Recently, Thrasher, *et al*, reported comparison of transvesical and extravesical and found complication rates of 9.4% and 3.7%, respectively.[16] A survey by Gibbonsin included 1000 transplant patients who were operated using Barry technique and found a 2.1% complication rate including bleeding, extravasations, and Vesicoureteral Reflux.[17] Conlin, *et al*, [18] reported a rate of 3% for grade 1 vesico-ureteral reflux (VUR) without stent and 46% with stent in the extravesical ureteroneocystosomy with Barry technique. Guitierrez [19] studied 79 transplant patients using extravesical Taguchi ureteroneocystosomy and reported two patients with fistula and but no VUR nor stenosis. They, therefore recommended extravesical technique because of its simplicity and low complication rate.

Advantages of extravesical ureteroneocystostomy include less operative time, avoidance of a separate cystostomy, virtually no hematuria, ability to use short ureters, no need for splints or stents, shortened Foley catheter drainage, and no interference with native ureteral function.[20] 

Significant shifts in the incidence of complications become apparent with the increasing use of stent. Several authors advocate the routine use of ureteral stent and maintain that which would result in a lower incidence of urological complications particularly urinary leaks and early postoperative obstructions [21, 22]. This was confirmed in a randomized study conducted by Benoit, *et al* [23]. However, the use of stents involves potential hazards including degradation of the stent, encrustation, migration and increased incidence of UTI [22].

For better results, many technical improvements took place in ureteroneocystostomy method and several authors introduced new techniques with lower complications and better outcomes. 

In 1993, Caparrós, *et al*, for the first time, used the combined techniques of Barry and Taguchi. The complication rate was about 12.8%. They reported the rate of ureteral stenosis as 3.9%, fistula 3.9% and complicated hematuria 4.9% [28]. For the first time, we used this technique in our institute and compared its complication with Barry technique which had been used in our center routinely.

Urine leaks and urinoma are relatively rare com­plications of transplantation and usually consti­tute an early postoperative problem. Extravasations of urine may occur from the renal pelvis, ure­ter, or ureteroneocystostomy site due to ureteral necrosis caused by vascular insufficiency or in­creased urinary pressures caused by obstruction. Early detection and repair have been in­strumental in reducing patient mortality. Antegrade pyelography is necessary to provide detailed information about the site and origin of the urinoma and in planning the appropriate intervention [24]. Small urine leaks may be treated with percuta­neous nephrostomy and stent placement. Caliceal leakage caused by infarction is treated with percu­taneous nephrostomy alone. Ureteral stents must be kept in place for 6–8 weeks after cessation of leakage to allow complete healing of the ureter and to preserve long-term patency [24]. 

We observed almost equal leakage rate in both studied groups caused probably due to fistula in Barry techniques 1%); no difference was found and the outcomes were comparable to other studies.[15-18] If the healing is unsuccessful, reimplantation of the ureter may be required. If distal necrosis is present, pyelocystostomy or pyeloureterostomy with the native ureter is performed. We had no morbidity in our study.

Urinary obstruction occurs in approximately 2% of transplantations and almost always within the first six months of the procedure.[25] Obstruction of the transplanted kidney may occur at any location but is most often happens at the site of implantation of the ureter into the bladder. Narrowing at the ureterovesical junction may be caused by scarring secondary to ischemia or rejection, by technical error during the ureteroneocystostomy or by kinking. These events account for more than 50% of obstructions that cause ureteral stricture. Less common causes of ureteral stricture include pelvic fibrosis, calculi, papillary necrosis, fungus ball, clots, and compression from an ex­trinsic mass such as adjacent peritransplant col­lections. Occasionally, obstruction that develops years after transplantation—especially in patients who had undergone multiple procedures—may be related to adhesions, vascular insufficiency, or fibrosis.[25,26] Strictures of the middle and upper ureters are mostly resulted from isch­emic or periureteral fibrosis and may require sur­gical interventions. However, ureteroureterostomy or pyeloureterostomy may be performed [27]. In this survey, we encountered no significant difference between the two groups in terms of consequences the rate of which was almost equal to that of other studies using Barry technique. The most common place for stenosis and obstruction was ureterovesical junction in our study. 

Post-transplantation complicated hematuria requiring endoscopic or surgical treatment in extravesical ureteral reimplantation is rarer than in intravesical technique. In our trial, complicated hematuria was not observed at all. We just found some degrees of simple hematuria which resolved by conservative management. 

We found that the Barry-Taguchi ureteroneocystostomy is as safe as the previous techniques which had been used in our institute and its complication rates are acceptable in comparison to other techniques. We also found that the time takes for ureteroneocystostomy is significantly lesser than the Barry technique, because there is no need for suturing the edges of the spatulated ureter to the bladder. It is obviously an easier technique with acceptable complication rates and outcome and based on these data, we substituted other techniques with it in our center.
